# A full-length recombinant *Plasmodium falciparum* PfRH5 protein induces inhibitory antibodies that are effective across common *PfRH5* genetic variants

**DOI:** 10.1016/j.vaccine.2012.10.106

**Published:** 2013-01-02

**Authors:** Leyla Y. Bustamante, S. Josefin Bartholdson, Cecile Crosnier, Marta G. Campos, Madushi Wanaguru, Chea Nguon, Dominic P. Kwiatkowski, Gavin J. Wright, Julian C. Rayner

**Affiliations:** a Malaria Programme, Wellcome Trust Sanger Institute, Wellcome Trust Genome Campus, Hinxton, Cambridge CB10 1SA, United Kingdom; b Cell Surface Signalling Laboratory, Wellcome Trust Sanger Institute, Wellcome Trust Genome Campus, Hinxton, Cambridge CB10 1SA, United Kingdom; c National Center for Parasitology, Entomology and Malaria Control, Phnom Penh, Cambodia

**Keywords:** Malaria, Vaccine, Erythrocyte invasion, Blood stage

## Abstract

The lack of an effective licensed vaccine remains one of the most significant gaps in the portfolio of tools being developed to eliminate *Plasmodium falciparum* malaria. Vaccines targeting erythrocyte invasion – an essential step for both parasite development and malaria pathogenesis – have faced the particular challenge of genetic diversity. Immunity-driven balancing selection pressure on parasite invasion proteins often results in the presence of multiple, antigenically distinct, variants within a population, leading to variant-specific immune responses. Such variation makes it difficult to design a vaccine that covers the full range of diversity, and could potentially facilitate the evolution of vaccine-resistant parasite strains. In this study, we investigate the effect of genetic diversity on invasion inhibition by antibodies to a high priority *P. falciparum* invasion candidate antigen, *P. falciparum* Reticulocyte Binding Protein Homologue 5 (PfRH5). Previous work has shown that virally delivered PfRH5 can induce antibodies that protect against a wide range of genetic variants. Here, we show that a full-length recombinant PfRH5 protein expressed in mammalian cells is biochemically active, as judged by saturable binding to its receptor, basigin, and is able to induce antibodies that strongly inhibit *P. falciparum* growth and invasion. Whole genome sequencing of 290 clinical *P. falciparum* isolates from across the world identifies only five non-synonymous *PfRH5* SNPs that are present at frequencies of 10% or more in at least one geographical region. Antibodies raised against the 3D7 variant of PfRH5 were able to inhibit nine different *P. falciparum* strains, which between them included all of the five most common PfRH5 SNPs in this dataset, with no evidence for strain-specific immunity. We conclude that protein-based PfRH5 vaccines are an urgent priority for human efficacy trials.

## Highlights

► PfRH5 is essential for *Plasmodium falciparum* erythrocyte invasion. ► Antibodies raised against full-length functional PfRH5 potently blocked invasion. ► Five common *PfRH5* polymorphisms were identified across 290 clinical isolates. ► Antibodies raised against one variant inhibited all other common PfRH5 variants. ► Correctly folded recombinant PfRH5 is a strong *P. falciparum* vaccine candidate.

## 1 Introduction

The development of an effective *Plasmodium falciparum* vaccine is the topic of intense research. Rational arguments can be advanced to support vaccines that target pre-erythrocytic or erythrocytic stages in humans, or parasite development within the mosquito [1]. The number of stages that could be targeted is compounded by the variety of potential approaches [2], which range from genetically or physically attenuated parasites [3], to subunit-based vaccines, of which there are hundreds of theoretical targets within the genome [4]. Regardless of the approach, however, the fundamental requirement for an effective malaria vaccine is the same – to effectively protect the largest number of people against the widest possible range of *P. falciparum* genetic variants.

Vaccines targeting the erythrocytic stage have their origins in the observations that passive transfer of immunoglobulins from immune to non-immune individuals markedly reduced parasitemia [5], and that immune adults directly inoculated with blood stage parasites avoid clinical symptoms, emphasizing the protective effect of antibodies targeting blood-stage antigens [6]. Several *P. falciparum* proteins have been suggested as potential blood-stage vaccine antigens, either because of their function, localization on the merozoite surface, or because data from immuno epidemiological studies suggest that they are targets of protective immunity [7,8]. All face the same fundamental challenge, however; by being directly exposed to the human immune system during natural infections, they are under considerable selective pressure, which can result in high levels of polymorphism [9], meaning that vaccine-induced immunity must be able to protect against multiple genetic variants. Although this is a significant challenge, the availability of genome sequences for hundreds of *P. falciparum* isolates [10] now makes it possible to measure the true scale of the challenge by identifying for each antigen which residues are variable, and in which populations.

*P. falciparum* Reticulocyte Binding Protein Homologue 5 (PfRH5) is a member of the super family of erythrocyte ligands referred to as the Reticulocyte Binding Like proteins (RBLs), at least one member of which is found in every *Plasmodium* genome [11]. PfRH5 differs from the other members of the PfRH family in that it is much smaller and the gene encoding PfRH5is refractory to genetic deletion in all tested strains, implying that it is essential for blood-stage growth [12]. PfRH5 bindserythrocytes and is implicated in the species tropism of erythrocyte invasion [12–14], and our laboratories have recently established that the receptor for PfRH5 is basigin/CD147 [15]. Of key importance for vaccine development is that unlike all other known erythrocyte–merozoite receptor–ligand interactions, the basigin-PfRH5 interaction was essential for erythrocyte invasion in every *P. falciparum* strain tested to date [15].

Its role in a universally required merozoite–erythrocyte interaction clearly highlightsPfRH5 as a promising vaccine target. However, early studies using antibodies raised against sub fragments of recombinant PfRH5 expressed in *E. coli* showed no [14] or very limited [12] efficacy in blood stage growth inhibition assays (GIAs). By contrast, immunization with recombinant viruses encoding full-length PfRH5 produced antibodies that cross-protected against multiple *P. falciparum* strains [16] suggesting that the conformation of the PfRH5 protein used as an antigen was critical for this activity. Here, we set out to establish whether full-length, functional, PfRH5 protein could induce growth-inhibiting antibodies. At the same time, we performed a systematic investigation of the impact of genetic diversity on invasion inhibitory activity, by testing anti-PfRH5 antibodies against *P. falciparum* strains that contained all common genetic variants of *PfRH5*, as defined in sequencing study of 227 *P. falciparum* clinical isolates [10]. The data has important implications for the vaccine candidacy of PfRH5.

## 2 Materials and methods

### 2.1 Parasite sequence variant analysis

Single nucleotide polymorphisms (SNPs) and allele frequencies were obtained from a published analysis of 227 clinical *P. falciparum* infections [10]. MalariaGen SNP catalogue release 1.0 data was used for all analysis (www.malariagen.net/data). PfRH5 sequence was verified for all strains used (see Supplement).

### 2.2 Protein expression plasmids, production and purification

Expression of the *P. falciparum* 3D7 PfRH5 ectodomain was described previously [15], and 7G8 and GB4 PfRH5 variants and 3D7 AMA1 were made and expressed according to the same principles. The basigin-L ectodomain was expressed as a soluble Cd4d3+4-hexa-his tagged protein, as described [17]. All proteins were produced as secreted proteins by the human HEK293E cell line grown in suspension and transiently transfected at a density of 5x10^5^ cells per mL as described [17,18]. His-tagged proteins were purified from spent supernatants after transient transfections on HisTrap™ HP columns (GE Healthcare) using an ÄKTAxpress™ (GE Healthcare).

### 2.3 Surface plasmon resonance

Surface plasmon resonance (SPR) studies were performed using a BIAcore™ T100 (GE Healthcare). Monomeric biotinylated bait proteins were immobilized on a streptavidin-coated sensor chip (GE Healthcare) and increasing concentrations of purified basigin was injected at 20 μl/min to determine equilibrium measurements or 100 μl/min for kinetic parameters. Both equilibrium and kinetic binding data were analyzed in the manufacturer's BIAcore™ T100 evaluation software (GE Healthcare).

### 2.4 Enzyme-linked immunosorbant assay (ELISAs)

Monobiotinylated proteins were captured on streptavidin-coated plates (Nunc) and then incubated for 90 min with 1 μg/ml primary antibody from which anti-Cd4 reactivity had been removed by preadsorbing with Cd4 protein. Alkaline phosphatase-linked secondary antibodies and appropriate detection reagents (Sigma) were used, and optical density measurements taken at 405 nm (Pherastar plus, BMG laboratories).

### 2.5 Antibodies

Polyclonal antisera against 3D7 PfRH5 and AMA1 were raised commercially and in parallel (Cambridge Research Biochemicals, Billingham, UK; immunization regime listed in Supplement). The use of animals to raise antisera was performed according to UK Home Office governmental regulations and approved by the local Sanger Institute ethical review board. Total IgG from the sera were purified on Hi-Trap™ Protein G HP columns (GE Healthcare) according to the manufacturer's instructions.

### 2.6 *P. falciparum* culture and invasion assays

*P. falciparum* parasites were cultured in human O+ erythrocytes at 5% hematocrit. Field strains were confirmed to contain only a single strain using PfMSP1 and PfMSP2 genotyping. Use of erythrocytes and serum from human donors was approved by the NHS Cambridgeshire 4 Research Ethics Committee, and all donors supplied written informed consent. For invasion assays, synchronized parasites were incubated in the presence or absence of antibodies for 24 h. Invasion assays were carried out in 96 well plates, with 3 identical wells set up for each strain/antibody concentration combination. Cultures were then fixed, and parasitized erythrocytes counted using SYBR Green I (Invitrogen) and flow cytometry, as described previously [19]. Invasion efficiency was calculated by comparing invasion in the presence of a given antibody concentration to invasion in the absence of antibodies.

## 3 Results

### 3.1 Genetic diversity in PfRH5 is limited

Whole genome sequencing by the MalariaGen consortium of 227 *P. falciparum* clinical isolates (125 samples from Africa (Mali, Burkina Faso and Kenya), 81 samples from Southeast Asia (Thailand and Cambodia) and 21 samples from Papua New Guinea [10]) identified fourteen polymorphisms in the *PfRH5* gene, two of which are synonymous and twelve non-synonymous (Table 1). This excess of non-synonymous over synonymous mutations is consistent with the overall trend of mutation rates in the *P. falciparum* genome and the specific increase in non-synonymous substitutions in proteins exposed on the merozoite surface [20]. Of the twelve non-synonymous mutations, only five SNPs reach a frequency greater than 10% in at least one population (Y147H, H148D, S197Y, C203Y and I410 M; marked * in Table 1), and can be considered the most common global variants in *PfRH5* that have been currently identified. Of these five SNPs, three are found in previously sequenced laboratory-adapted *P. falciparum* strains, but Y147H and H148D, which reach 30% frequency in Southeast Asian clinical isolates, are not present in any lab strain sequenced to date (Table 1). We identified two isolates from Cambodia that contained these mutations for use in this study. PfRH5 sequences from all strains was confirmed by sequencing, and the full haplotype of each strain is shown in Supplementary Table 1.

### 3.2 Expression and purification of biochemically active full-length recombinant PfRH5 protein variants

The chances of raising an effective polyclonal antibody response are likely to be significantly higher if the recombinant antigen closely mimics the conformation of the native antigen [21]. Previously, we have shown that biochemically active PfRH5 can be expressed as a secreted full-length protein using a mammalian expression system [15]. The 3D7 variant of PfRH5 was used in these initial experiments, which is notable because it contains six cysteine residues of which C203 is a relatively rare variant, meaning most strains express PfRH5 with five cysteines (Table 1). As sulfhydryl groups of cysteines are normally oxidized to form structurally critical disulfide bonds in secreted proteins, polymorphism at C203 suggests that cysteine residues in some variants of PfRH5, most likely at positions 203 and 329, are unpaired [12]. To determine whether PfRH5 variants that contain five cysteines were biochemically active, we expressed 7G8 and GB4 variantsin HEK239E cells as Cd4d3+4-tagged monobiotinylated “bait” proteins [17]. Cells were transiently transfected, and all variants produced 2–10 μg of protein per ml of cell culture supernatant 6 days post-transfection. These yields were produced using a cost-effective transfection approach, which typically resulted in a transfection efficiency of ∼40%, and could presumably be increased in stably expressing isolates.

**Fig. 1:**
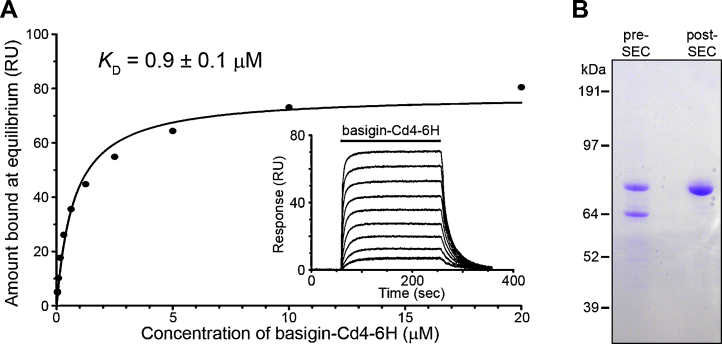
Surface plasmon resonance demonstrates biochemical activity of recombinant full-length 7G8 PfRH5-Cd4. (A) 7G8 PfRH5-Cd4-biotin was specifically captured in a flow cell of a streptavidin-coated chip using an approximate molar equivalent of Cd4-biotin immobilized in a neighbouring flow cell used as a reference. Serial dilutions of purified basigin-Cd4-6H were injected until equilibrium was reached (solid bar, inset) and reference-subtracted data were used to plot the binding responses once equilibrium had been achieved. An equilibrium dissociation constant was calculated using non-linear regression fitting of a simple Langmuir binding isotherm to the data (solid line). Note that the binding was saturable and therefore specific and a *K*_D_ of 0.9 ± 0.13 μM (mean ± SEM) was calculated from two independent experiments (see Supplementary Table 2). A representative experiment using 7G8 is shown, equilibrium constants for 3D7 and GB4 were calculated in the same way and are reported in Supplementary Table 2. (B) 3D7 PfRH5-Cd4-d3+4-6H was purified using Ni^2+^-NTA-Sepharose and resolved as two bands (∼85 and 65 kDa) using using Coomassie staining after SDS-PAGE under reducing conditions. The 85 kDa protein, which is consistent with the expected size of the full-length PfRH5-Cd4-d3+4-6 H protein, could be separated from the smaller 65kDa band using size exclusion chromatography (post-SEC).

To test variants for biochemical activity, we expressed and purified the extracellular region of human basigin as a soluble Cd4d3+4-6H-tagged protein and used surface plasmon resonance to quantify interaction affinities. Serial dilutions of basigin were injected over PfRH5 immobilized on a sensor chip until equilibrium was reached (Fig. 1A). Saturable binding was observed, and the biophysical binding parameters were very similar in all cases (Supplementary Table 2). The association (*k*_a_) and dissociation (*k*_d_) kinetic rate constants were in good agreement with the equilibrium analysis (Supplementary Table 2), providing an independent measure of the interaction parameters. The *K*_D_ was essentially identical to that reported for the same experiment performed in the reciprocal orientation using purified 3D7 PfRH5 [15] suggesting that the proportion of biochemically active protein was high.

### 3.3 Generation of polyclonal antisera and testing against PfRH5 variants

**Fig. 2:**
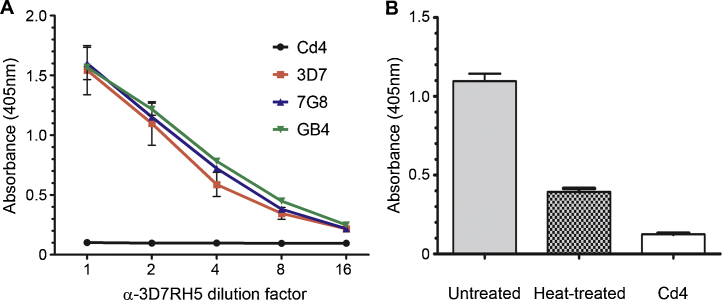
Immunoreactivities to recombinant PfRH5 7G8 and GB4 are indistinguishable from the 3D7 variant used as an antigen. (A) The 3D7, 7G8 and GB4 PfRH5 variants (colored lines, as indicated in the key) were expressed as Cd4-biotin tagged recombinant proteins, normalized, and specifically captured on a streptavidin-coated microtitre plate. Each variant was equally immunoreactive to a polyclonal antiserum that was raised using the 3D7 variant. Immunoreactivity to the Cd4 tag was removed by preadsorption of the antiserum against Cd4; the preadsorbed antiserum was shown to lack anti-Cd4 immunoreactivity (black line). (B) Recombinant PfRH5 contains heat-labile epitopes. The 3D7 variant of PfRH5-Cd4-biotin was again captured on a streptavidin-coated plate either without treatment or after denaturation by heat (80 °C, 10 min). The two proteins were tested for their immunoreactivity to the anti-RH5 polyclonal antiserum that had been preadsorbed against Cd4 (shown as a control). Data points are means; error bars represent SEM; *n* = 3.

To raise polyclonal antisera against full-length 3D7 PfRH5, we expressed and purified the protein as previously described [15]. Purified PfRH5-Cd4d3+4-6H runs as two bands at ∼85 and ∼65 kDa (Fig. 1B), which, when the size of the Cd4 tag (∼25 kDa) is taken into account, corresponds well to the two forms of native PfRH5 (63 and 45 kDa) observed in parasite cultures [12], suggesting the same proteolytic processing occurs when expressed in HEK293 cells as in the parasite. We were able to resolve the 85 kDa band from the 65 kDa form by size-exclusion chromatography (Fig. 1B). Purified PfRH5 protein showed no measurable loss of activity when stored at 4 °C for several months (unpublished observations). Rabbits were immunized with 1 mg of purified 3D7 PfRH5-Cd4d3+4-6H protein. To remove anti-Cd4 antibodies, the antisera were preadsorbed with a Cd4-tag-containing protein and shown to then lack anti-Cd4 immunoreactivity by ELISA (Fig. 2A). Preadsorbed total IgG was then used in ELISAs to quantify the immuno reactivity against both the immunizing 3D7 antigen, and cross-reaction with the 7G8 and GB4 variants. No difference in immuno reactivity to any of the three variants was observed (Fig. 2A). We heat-treated 3D7 PfRH5 and observed a loss of immuno reactivity suggesting that a good proportion of the antibodies recognized heat-labile conformational epitopes (Fig. 2B).

### 3.4 Antibodies raised against the 3D7 PfRH5 variant inhibit parasites that collectively include all currently identified common PfRH5 genetic variants

**Fig. 3:**
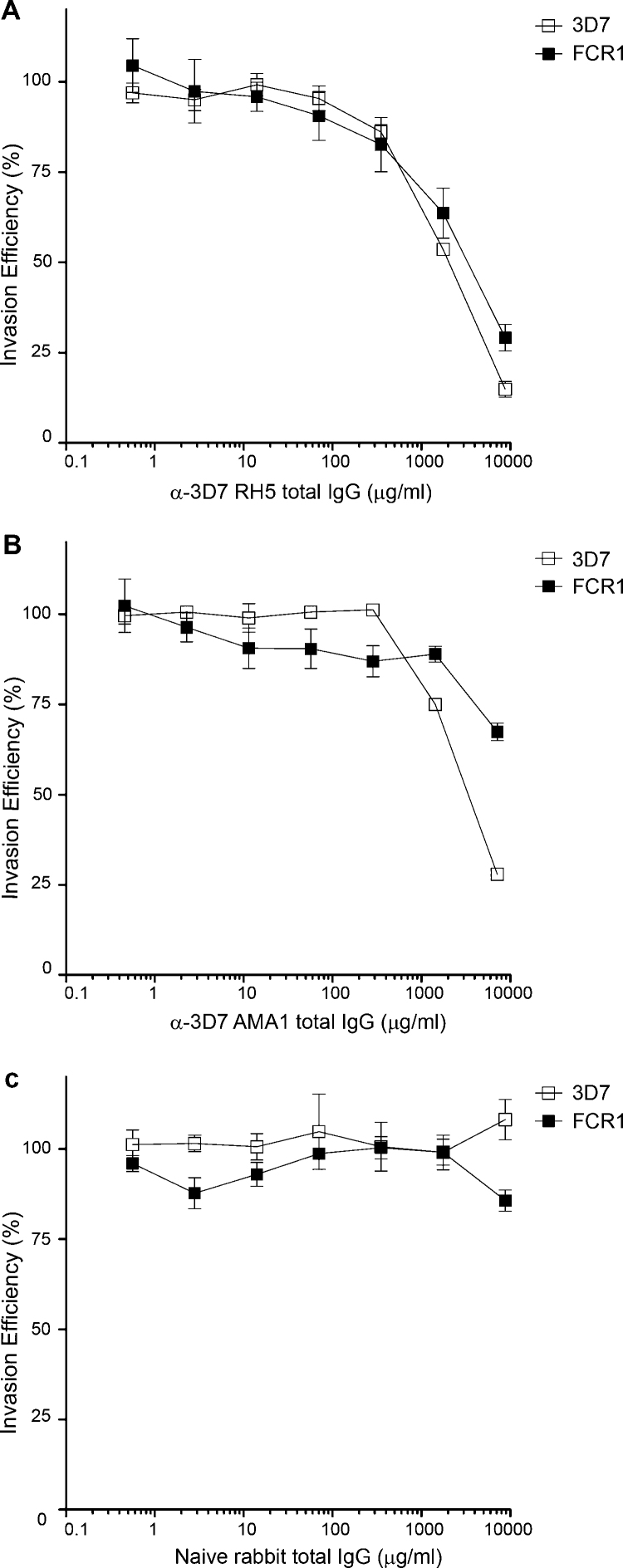
Polyclonal antibodies raised against 3D7 PfRH5 inhibit both 3D7 and FCR1 *P. falciparum* parasites. (A) Total IgG purified from a rabbit immunized with the 3D7 variant of PfRH5 was used in invasion assays with the 3D7 and FCR1 *P. Falciparum* strains. Growth inhibition was observed to a similar extent for both strains with an IC50 ratio for FCR1 of 1.6 (95% CI 1.17–2.18). (B) Total IgG purified from a rabbit immunized with the 3D7 AMA variant was used in erythrocyte invasion assays with the 3D7 or FCR1 *P. Falciparum* strains and showed strain-specific growth inhibition. (C) Total IgG purified from unimmunized rabbits was used in erythrocyte invasion assays with the 3D7 or FCR1 *P. falciparum* strains and had no inhibitory effect on erythrocyte invasion. Each IgG concentration/strain combination was assayed in three wells in a 96 well plate; data points are means of all three wells, with standard deviation error bars shown.

To establish whether antibodies raised against the full-length 3D7 variant of PfRH5 could inhibit erythrocyte invasion, total IgG was purified from anti-PfRH5 rabbit polyclonal serum and added in increasing concentrations to late trophozoite stage *P. falciparum* parasite cultures. Parasites were cultured in the presence of IgG for 24 hours before parasitemia was measured using a fluorescent DNA dye [19]. Anti-PfRH5 IgG had a clear dose-dependent inhibitory effect on the growth of 3D7 parasites, with an IC50 of 1.9 mg/ml total IgG (Fig. 3A). FCR1 was chosen for initial strain-specificity trials because it contains two of the five common *PfRH5* SNPs (Table 1). Dose–response curves for inhibition of FCR1 parasites were similar to those against 3D7, with an IC50 of 3.1 mg/ml total IgG. (Fig. 3A). By contrast, antibodies raised against the entire ectodomain of the 3D7 variant of AMA1 had a marked strain-specific effect, with only a 40% reduction in FCR1 growth even at the maximum concentration of 10 mg/ml (Fig. 3B), consistent with the known strain-specific properties of anti-AMA1 antibodies [22]. Neither strain was inhibited by control pre-immune rabbit total IgG (Fig. 3C).

**Fig. 4:**
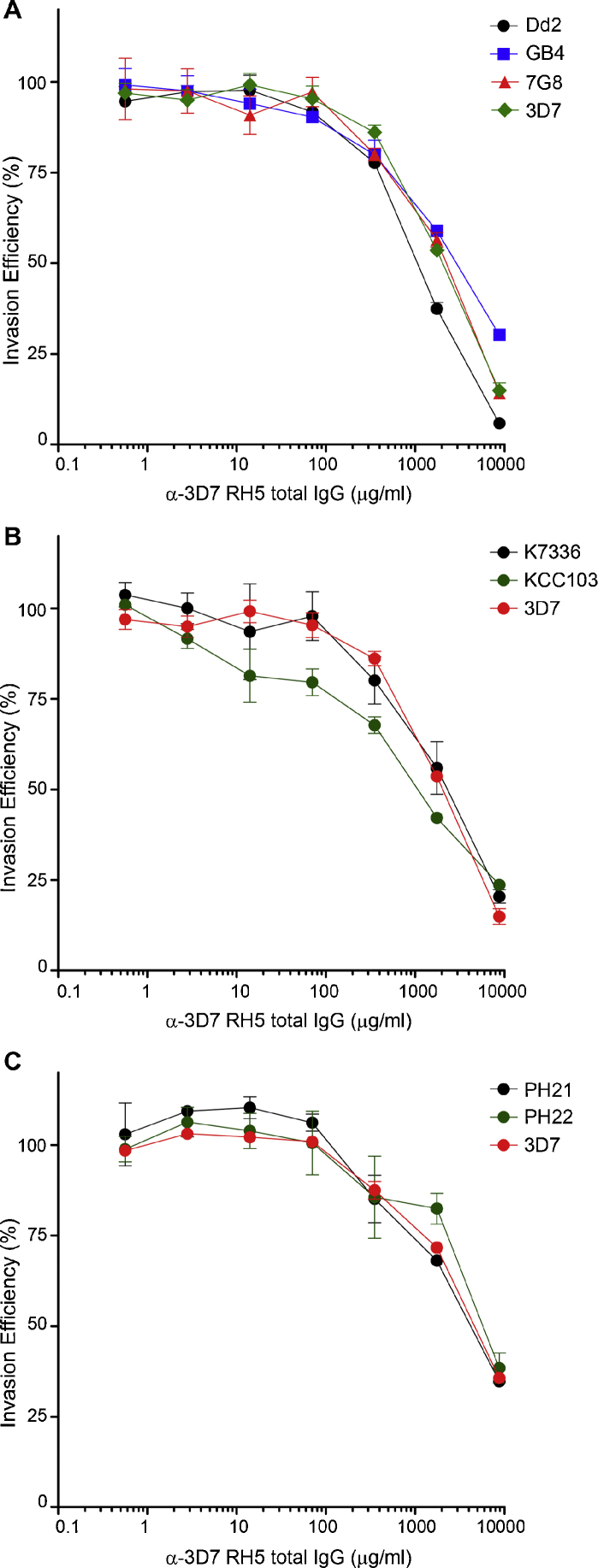
Polyclonal antibodies raised against 3D7 PfRH5 inhibit *P. falciparum* strains that collectively include all common PfRH5 variants. (A) Total IgG purified from a rabbit immunized with the 3D7 variant of PfRH5 was used in invasion assays with Dd2, 7G8, GB4 and 3D7*P*. *falciparum* strains. IC50 ratios were 0.5, 1.0 and 1.4 for Dd2, 7G8 and GB4, respectively. 95% CI for IC50s were 0.47–0.63 (Dd2), 0.71–1.36 (7G8) and 1.18–1.71 (GB4). (B) Total IgG purified from a rabbit immunized with the 3D7 variant of PfRH5 was used in erythrocyte invasion assays with two *P. falciparum* isolates from Kenya (K7336 and KCC103), and the 3D7 *P*. *falciparum* strain. IC50 ratios were 1.1 and 0.5 for K7336 and KCC103, respectively. 95% CI for IC50s were 0.81–1.42 (K7336) and 0.35–0.76 (KCC103). (C) Total IgG purified from a rabbit immunized with the 3D7 variant of PfRH5 was used in erythrocyte invasion assays with two *P. falciparum* isolates from Cambodia (PH21 and PH22) and the 3D7 *P. falciparum* strain. IC50 ratios were 0.8 and 1.3 for PH21 and PH22 respectively. 95% CI for IC50s were 0.59–1.35 (PH21) and 0.93–1.76 (PH22). Each IgG concentration/strain combination was assayed in three wells in a 96 well plate; data points are means of all three wells, with standard deviation error bars shown.

To more comprehensively test the inhibitory effect of antibodies raised against the 3D7 variant of PfRH5, we tested seven additional *P. falciparum* isolates, that between them included *PfRH5* sequences that encompass the five common *PfRH5* SNPs. The effect of anti-PfRH5 on 3D7 was re-measured in every replicate as a positive control. The dose-response curves for laboratory (Fig. 4A), Kenyan (Fig. 4B) and Cambodian strains (Fig. 4C) were broadly comparable with that of 3D7. As expected, the inhibitory effect varied slightly between independent experiments due to biological variation, such as different starting growth rates. To enable a quantitative comparison between experiments, the IC50 for each test strain was compared to that of the 3D7 positive control reference strain performed in parallel. IC50 ratios are listed in the figure legends, and range from a low of 0.53 (Kenya KCC103) to a high of 1.6 (FCR1). Statistical analysis (*f*-test) established that no IC50 represents a significant variation from 3D7 (*p* = 0.623). Serum from a second rabbit immunized with full-length 3D7 PfRH5 were also tested and showed no significant difference in growth inhibition between 3D7 and FCR1 parasites (data not shown). Antibodies raised against the 3D7 PfRH5 variant therefore have broad inhibitory activity across all currently identified common genetic variants, with no evidence for significant variant-specificity.

## 4 Discussion

The essentiality of the PfRH5–basigin interaction for erythrocyte invasion across all strains tested [15] have highlighted PfRH5 as a particularly promising blood-stage vaccine candidate. The development of subunit malaria vaccines has, however, been partly limited by the technical difficulties of expressing biochemically active *Plasmodium* proteins in heterologous expression systems [23]. While viral delivery systems could be considered [24], blood-stage vaccines based on proteins could be more easily combined with vaccines targeting other stages of the *Plasmodium* lifecycle such as RTS, S [25]. Using a mammalian expression system, we have been able to produce full-length PfRH5 protein and unambiguously demonstrate its biochemical activity by showing saturable binding to its receptor, basigin [15]. While other assays such as erythrocyte binding and immuno reactivity to parasite-exposed sera have traditionally been used to validate the function of recombinant parasite proteins, these assays might not be sufficient to assess correct folding and biochemical activity, which are well-recognized prerequisites of good vaccine antigens. The identification of basigin as the erythrocyte PfRH5 receptor now enables the unequivocal testing of the biochemical activity of any recombinantly produced PfRH5 in a quantifiable manner. It remains to be seen whether recombinant RH5 protein produced either using *E. coli* with a refolding protocol [12], a wheat germ lysate system [26], or used chemically synthesized peptides [27], has this confirmed biochemical activity.

Using a recombinant PfRH5 based on the 3D7 sequence of *P. falciparum*, we were able to raise antibodies that have growth inhibitory effects across multiple *P. falciparum* strains. Genetic diversity in blood-stage vaccine candidates is a concern because of the risk of raising allele-specific antibody responses, and such responses have been observed for multiple *P. falciparum* merozoite proteins, including those advanced for human trials such as MSP1 [28], MSP3 [28–30] and AMA1 [22]. Interrogation of the whole genome sequences of 227 clinical *P. falciparum* isolates [10] showed that the gene encoding PfRH5 has only a few SNPs present at high frequency. This might be due to the fact that PfRH5 is not a target of natural humoral immunity since, remarkably, sera from Kenyan adults are only weakly immunoreactive to PfRH5 [16]. However, even single polymorphisms can lead to immune evasion, as exemplified by AMA1, where a few high-frequency variants can have a major effect on the specificity of the immune response [31]. However, our study shows that at least *in vitro*, no currently identified common PfRH5 variant has a significant effect on the ability of anti-PfRH5 IgG to inhibit parasite growth. While the SNPs tested here are based on the largest published whole *P. falciparum* genome sequence analysis, additional high frequency PfRH5 SNPs may be identified as more samples are sequenced. However, preliminary analysis of more than 1500 global clinical isolates does not reveal any additional PfRH5 SNPs present at higher than 0.10 non-reference allele frequency in any geographic region (Malaria GEN unpublished data). The five SNPs investigated here may therefore represent the most common globally distributed PfRH5 polymorphisms that can be identified using current variation calling pipelines.

Interestingly, previous sequencing of laboratory-adapted isolates [13] identified eight additional PfRH5 SNPs (E48K, I204R, I204K, N347Y, N347D, Y358F, E362D, K429N) that have so far not been seen in any clinical samples, all in strains with an increased ability to invade *Aotus* erythrocytes [13]. It remains to be seen whether these SNPs have arisen in laboratory strains spontaneously during *in vitro* culture, or whether they are present in natural isolates at very low frequencies. However, what is clear is that *P*. *falciparum* strains containing any of these eight SNPs would be non-representative choices for vaccine challenges or antigen design. In fact the 3D7 PfRH5 sequence would also be a non-representative choice, because it contains the C203Y variant which is relatively rare in field isolates.

In summary, using a mammalian expression system, we have produced full-length biochemically active recombinant PfRH5 that can induce inhibitory antibodies to *P. falciparum* strains expressing all the currently identified common variants of *PfRH5*. Broad cross-inhibitory activities of anti-PfRH5 antibodies have previously been demonstrated using virally delivered immunogen [16]. Our study adds significant new data to the vaccine candidacy of PfRH5, by showing that the same strain-transcendent responses can be induced using a protein immunogen, and by extending the range of variants tested. There is therefore now a strong rationale to advance this candidate to Phase I/IIa human trials, which, if successful, could be combined with vaccines targeting other stages of the *P. falciparum* life cycle, such as the sporozoite stage in the RTS, S, vaccine currently undergoing Phase III trials in Africa [25].

## Figures and Tables

**Table 1:** Global allele frequencies of PfRH5 sequence variants in clinical isolates.

AA	3D7	Nref	Freq Africa	Freq Asia	Freq PNG	Lab strain
88	N	D	0.01	0.00	0.00	–
147*	Y	H	0.09	0.28	0.05	–
148*	H	D	0.10	0.30	0.05	–
197*	S	Y	0.00	0.54	0.38	FCR1
203*	C	Y	0.79	0.62	0.9	FCR1, GB4
233	A	E	0.00	0.00	0.05	–
365	H	N	0.01	0.00	0.00	–
371	V	I	0.05	0.00	0.00	–
407	I	V	0.03	0.00	0.00	GB4
410*	I	M	0.00	0.35	0.10	Dd2
477	Q	H	0.01	0.00	0.00	–
493	I	V	0.00	0.00	0.00	–

Publically available aggregate data from the sequencing of 227 *P. falciparum* clinical isolates by the MalariaGen consortium [10] was searched for single nucleotide polymorphisms (SNPs) in *PfRH5*. Only SNPs that cause a change in amino acid (AA) at a specific position relative to the reference *P. falciparum* sequence (3D7), are included and are referred to as non-reference SNPs (Nref) in the v1.0 SNP catalogue. The allele frequency for each non-ref PfRH5 SNP in the 125 African, 81 Asian and 21 Papua New Guinean (PNG) samples are listed above. SNPs present in at least 10% of at least one population are judged to be common, and are indicated by an asterisk ‘*’. SNPs identified in previously published *PfRH5* sequences for laboratory-adapted *P. falciparum* isolates [13] are listed. Note that most naturally varying SNPs have not yet been identified in laboratory strains.
