# Supplementary material for: A full-length recombinant Plasmodium falciparum PfRH5 protein induces inhibitory antibodies that are effective across common PfRH5 genetic variants
Source: Vaccine. 2013 Jan 2;31(2):373–9. doi: 10.1016/j.vaccine.2012.10.106 (PMC3538003; doi:10.1016/j.vaccine.2012.10.106)
Supplement: Supplementary file 1 [file mmc1.docx]

**Supplementary Materials and Methods**

**Protein expression plasmids, production and purification**

The entire predicted ectodomain regions of PfRH5 and AMA1 were flanked by unique NotI and AscI sites and cloned into a protein expression vector that included an N-terminal high-scoring signal peptide from a mouse immunoglobulin kappa light chain and a C-terminal tag consisting of the rat Cd4 domains 3 and 4 tag followed by a hexa-his tag [[1](#_ENREF_1), [2](#_ENREF_2)]. All the threonines and serines present within N-linked glycosylation sequons were systematically mutated to alanine to prevent inappropriate glycosylation. Monomeric monobiotinylated PfRH5 variants were prepared by subcloning the NotI/AscI flanked extracellular regions into a vector containing a C-terminal biotinylatable tag which could be enzymatically biotinylated during expression by the coexpression of a secreted form of the protein biotin ligase, BirA [[3](#_ENREF_3)]. All ectodomains were codon optimised for mammalian expression and chemically synthesized (Geneart AG, Regensburg, Germany).

**Surface plasmon resonance**

For surface plasmon resonance (SPR) studies approximately 150 RU of the negative control “tag-alone” bait (biotinylated rat Cd4d3+4) were immobilised in the reference flow cell and the approximate molar equivalents of the test proteins were captured in the other flow cells. Purified basigin-L-Cd4d3+4-6H was separated by size exclusion chromatography on a Superdex™ 200 Tricorn™ 10/600 column (GE Healthcare) in HBS-EP (GE Healthcare) just prior to use in SPR experiments to remove any protein aggregates that are known to influence kinetic measurements. Increasing concentrations of purified basigin was injected at 20 µl/min to determine equilibrium measurements or 100 μl/min for kinetic parameters. 2M NaCl was used as a regeneration solution at the end of each cycle. Duplicate injections of the same analyte concentration were performed in each experiment and were superimposable demonstrating no loss of ligand activity after regenerating the surface. Both equilibrium and kinetic binding data were analysed in the manufacturer’s BIAcore™ T100 evaluation software (GE Healthcare). Both the equilibrium and kinetic binding data were replicated using independent protein preparations of both ligand and analyte proteins. All experiments were performed at 37°C in HBS-EP.

**Antibodies**

1mg of protein was divided equally between immunisations and administered for the first boost with Freunds complete adjuvant as a subcutaneous, nodal area implant. Subsequent boosts were administered using Freunds incomplete adjuvant as subcutaneous dorsal injections.

**Enzyme-linked immunosorbant assay (ELISAs)**

Monobiotinylated proteins were captured on streptavidin-coated plates (Nunc) for up to one hour before being incubated for 90 minutes with 1 μg/ml primary antibody. To remove the anti-Cd4 reactivity in the polyclonal antisera, the antisera were preadsorbed against the Cd4 protein. Preadsorbed antisera were shown to lack immunoreactivity against Cd4 by ELISA, e.g. see Fig. 2A. The plates were washed in HBS/0.1% Tween-20 (HBST) before incubation with an appropriate secondary antibody conjugated to alkaline phosphatase (Sigma). Plates were washed 3x HBST and 1x HBS before adding 100 µl p-nitrophenyl phosphate (Sigma 104 alkaline phosphatase substrate) at 1 mg/ml. Optical density measurements were taken at 405 nm on a Pherastar plus (BMG laboratories).

***P. falciparum* culture and invasion assays**

3D7, FCR1, Dd2, 7G8 and GB4 parasites were obtained from MR4 (www.mr4.org); K7336 and KCC103 were a kind gift from the Malaria Immunology Group at KEMRI-Kilifi. PH21 and PH22 were a kind gift from Dr. Arjen Dondorp and Dr. Rupam Tripura at the Mahidol-Oxford Tropical Medicine Research Unit, and culture adapted at the Sanger Institute. The PfRH5 gene was amplified and sequenced from all parasite strains used in this study to confirm previously published SNPs, using primers Rh5-F (5’- ATGATAAGAATAAAAAAAAAATTAATTTTGACCATT-3’) and Rh5-R (5’- TCATTGTGTAAGTGGTTTATTTTTTTTATATGTTTG-3’).

All parasites were cultured in in complete medium (RPMI-1640 containing 10% human serum), under an atmosphere of 1% O_2_, 3% CO_2_, and 96% N2. Invasion assays were carried out using synchronized in round-bottom 96-well plates, with a culture volume of 100 µL per well at a hematocrit of 2%. Before invasion assays were set up, parasites were synchronized at early stages with 5% (w/v) D-sorbitol (Sigma), then trophozoite stage parasites were incubated in the presence or absence of antibodies for 24 hours at 37 °C inside a static incubator culture chamber (VWR), gassed with 1% O_2_, 3% CO_2_, and 96% N_2_. All antibodies were dialysed into RPMI before addition into invasion assays. After invasion, parasites were detected using 1:5,000 SYBR Green I (Invitrogen). SYBR Green I stained samples were excited with a 488 nm UV laser (20 mW) on a BD Calibur flow cytometer (BD Biosciences) and detected with a 530/30 filter. BD FACS Diva software (BD Biosciences) was used to analyse 50,000 events for each sample. FSC and SSC voltages of 423 and 198, respectively, and a threshold of 2,000 on FSC were applied to gate the erythrocyte population. The data collected were further analyzed with FlowJo (Tree Star). GraphPad Prism (GraphPad Software) was used to plot the parasitemia data.

**References**

[1] Brown MH, Barclay AN. Expression of immunoglobulin and scavenger receptor superfamily domains as chimeric proteins with domains 3 and 4 of CD4 for ligand analysis. Protein engineering 1994 Apr;7(4):515-21.

[2] Crosnier C, Staudt N, Wright GJ. A rapid and scalable method for selecting recombinant mouse monoclonal antibodies. BMC biology 2010;8:76.

[3] Sun Y, Gallagher-Jones M, Barker C, Wright GJ. A benchmarked protein microarray-based platform for the identification of novel low-affinity extracellular protein interactions. Analytical biochemistry 2012 May 1;424(1):45-53.

[4] Hayton K, Gaur D, Liu A, Takahashi J, Henschen B, Singh S, et al. Erythrocyte binding protein PfRH5 polymorphisms determine species-specific pathways of Plasmodium falciparum invasion. Cell host & microbe 2008 Jul 17;4(1):40-51.

[5] Manske M, Miotto O, Campino S, Auburn S, Almagro-Garcia J, Maslen G, et al. Analysis of Plasmodium falciparum diversity in natural infections by deep sequencing. Nature 2012 Jun 13.

| **AA** | **3D7** | **Nref** | **FCR1** | **Dd2** | **GB4** | **7G8** | **K7336** | **KCC103** | **PH21** | **PH22** |
| --- | --- | --- | --- | --- | --- | --- | --- | --- | --- | --- |
| **88** | N | ***D*** | N | N | N | N | N | N | N | N |
| **147*** | Y | ***H*** | Y | Y | Y | Y | Y | Y | ***H*** | ***H*** |
| **148*** | H | ***D*** | H | H | H | H | H | H | ***D*** | ***D*** |
| **197*** | S | ***Y*** | ***Y*** | S | S | S | S | S | ***Y*** | ***Y*** |
| **203*** | C | ***Y*** | ***Y*** | C | ***Y*** | ***Y*** | ***Y*** | C | ***Y*** | ***Y*** |
| **233** | A | ***E*** | A | A | A | A | A | A | A | A |
| **365** | H | ***N*** | H | H | H | H | H | H | H | H |
| **371** | V | ***I*** | V | V | V | V | V | V | V | V |
| **407** | I | ***V*** | I | I | ***V*** | I | I | I | I | I |
| **410*** | I | ***M*** | I | ***M*** | I | I | I | I | I | I |
| **477** | Q | ***H*** | Q | Q | Q | Q | Q | Q | Q | Q |
| **493** | I | ***V*** | I | I | I | I | I | I | I | I |

**Supplementary Table 1: PfRH5 haplotypes of *P. falciparum* strains used in this study.**

The amino acid (AA) sequence for all strains used in this study was determined at each of the twelve non-ref non-synonymous SNPs (Nref), and Nref alleles are indicated by bold and italicization. SNPs present in at least 10% of at least one population are indicated by an asterisk. PfRH5 sequences for 3D7, FCR1, Dd2, GB4 and 7G8 and the Cambodian isolates PH21 and PH22 have been previously published[[4](#_ENREF_4), [5](#_ENREF_5)], and were confirmed by sequencing the strains used in this study (see Methods). PfRH5 sequences for K7336 and KCC103 were generated in this study.

| **PfRH5**  **variant** | **Expt.**  **#** | ***K*_D_**  **(µM)** | **fit error** | **Mean**  **(µM)** | **SEM** | ***k*_a_**  **(M^-1^s^-1^)** | **fit error** | **Mean**  **(M^-1^s^-1^)** | **SEM** | ***K*_d_**  **(s^-1^)** | **fit error** | **Mean**  **(s^-1^)** | **SEM** | **t½**  **(s)** | **Mean**  **(s)** | **SEM** |
| --- | --- | --- | --- | --- | --- | --- | --- | --- | --- | --- | --- | --- | --- | --- | --- | --- |
|  | 1 | 1.6 | ±0.1 |  |  | 89600 | ±200 |  |  | 0.24 | ±0.01 |  |  | 2.95 |  |  |
| **3D7** |  |  |  | 1.6 | 0 |  |  | 93000 | 3000 |  |  | 0.238 | 0.002 |  | 2.945 | 0.005 |
|  | 2 | 1.6 | ±0.1 |  |  | 95600 | ±100 |  |  | 0.236 | ±0.003 |  |  | 2.94 |  |  |
|  | 1 | 1.0 | ±0.2 |  |  | 127600 | ±200 |  |  | 0.256 | ±0.006 |  |  | 2.71 |  |  |
| **7G8** |  |  |  | 0.9 | 0.1 |  |  | 125000 | 3000 |  |  | 0.251 | 0.006 |  | 2.77 | 0.06 |
|  | 2 | 0.8 | ±0.1 |  |  | 121500 | ±200 |  |  | 0.245 | ±0.005 |  |  | 2.82 |  |  |
|  | 1 | 1.1 | ±0.2 |  |  | 118000 | ±300 |  |  | 0.216 | ±0.006 |  |  | 3.21 |  |  |
| **GB4** |  |  |  | 1.0 | 0.2 |  |  | 110000 | 10000 |  |  | 0.23 | 0.01 |  | 3.0 | 0.2 |
|  | 2 | 0.8 | ±0.2 |  |  | 95900 | ±200 |  |  | 0.244 | ±0.005 |  |  | 2.84 |  |  |

**Supplementary Table 2. PfRH5 from 3D7, 7G8 and GB4 bind human basigin with similar biophysical binding parameters.** Equilibrium and kinetic measurements were calculated from surface plasmon resonance studies using serial dilutions of basigin-Cd4-6H as an analyte and 3D7, 7G8 and GB4 PfRH5-Cd4-biotin as the immobilised ligands. Each experiment was performed twice (Expt. #). The parameters from each experiment are derived by globally fitting a simple 1:1 binding model to a family of binding curves produced from the dilution series of the basigin protein. Half-lives (t½ ) were calculated by ln2/*k*d.
